# Quantifying Interfacial Ion Transfer at Operating Potassium-Insertion Battery Electrodes within Highly Concentrated Aqueous Solutions

**DOI:** 10.1021/acsami.4c03645

**Published:** 2024-06-17

**Authors:** Zachary T. Gossage, Ryoichi Tatara, Tomooki Hosaka, Shinichi Komaba

**Affiliations:** Department of Applied Chemistry, Tokyo University of Science, Tokyo 162-8601, Japan

**Keywords:** scanning electrochemical microscopy, K-ion battery, redox, electrode interface, ion transfer

## Abstract

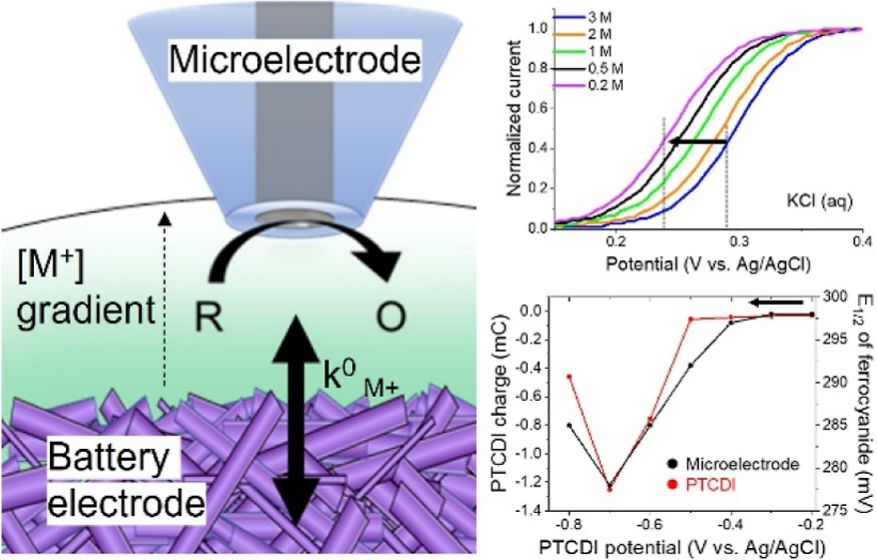

Electrode/electrolyte interfacial ion transfer is a fundamental process occurring during insertion-type redox reactions at battery electrodes. The rate at which ions move into and out of the electrode, as well as at interphase structures, directly impacts the power performance of the battery. However, measuring and quantifying these ion transfer phenomena can be difficult, especially at high electrolyte concentrations as found in batteries. Herein, we report a scanning electrochemical microscope method using a common ferri/ferrocyanide (FeCN) redox mediator dissolved in an aqueous electrolyte to track changes in alkali ions at high electrolyte concentrations (up to 3 mol dm^–3^). Using voltammetry at a platinum microelectrode, we observed a reversible *E*_1/2_ shift of ∼60 mV per decade change in K^+^ concentrations. The response showed high stability in sequential measurements and similar behavior in other aqueous electrolytes. From there, we used the same FeCN mediator to position the microelectrode at the surface of a potassium-insertion electrode. We demonstrate tracking of local changes in the K^+^ concentration during insertion and deinsertion processes. Using a 2D axisymmetric, finite element model, we further estimate the effective insertion rates. These developments enable characterization of a key parameter for improving batteries, the interfacial ion transfer kinetics, and future work may show mediators appropriate for molar concentrations in nonaqueous electrolytes and beyond.

## Introduction

Ion transfer at the electrode and electrolyte interface is a fundamental process that occurs during operation of batteries and other energy storage/generation devices.^1−3^ When devices like Li-ion batteries (LIBs) provide energy, both ion and electron transfer occur at the positive and negative lithium insertion materials. During de/insertion reactions at the electrode surfaces, the Li^+^-ion flux between an incorporated state within the electrode and solubilized ions dissociating within the polar solvent. The ions often need to transfer across highly complex interphase structures at the electrode–electrolyte interfaces, e.g., solid–electrolyte interphase (SEI) and cathode–electrolyte interphase.^4−7^ These interfacial ion transfer processes likely impact the rate performance and limitations of various battery chemistries.^8,9^ While ion transfer processes are an essential component to battery operation, simple quantitative methods for measuring the ion transfer process remain limited.^10,11^ Researchers tend to rely on bulk methodologies, such as impedance spectroscopy, to understand properties of the SEI.^8,9,12^ While those methods provide an evaluation of ion transfer resistances at the electrode, quantification of the actual rates via tracking of the ions during operation of the electrode is also highly appealing.^11^ Furthermore, developing an understanding of the connection between small, localized regions and the bulk electrode can provide more thorough insights for improving performance.^10,13−15^

In recent years, scanning electrochemical microscopy (SECM) has proven to be a powerful instrument for gaining localized dynamic information and mapping of various electrochemical processes within energy storage. This includes quantification of ion transfer,^11,16^ as well as insights on the interphase formation process,^15,17−19^ single particle measurements,^20^ gas evolution,^21^ among others.^13,15,22^ Although SECM is well suited for measuring localized ion transfer, most of the available ion sensing methods tend to show instability at typical battery electrolyte concentrations of ∼1 mol dm^–3^ (≡1 M) and above. There are some alternative methods, including scanning ion conductance microscopy,^23^ though they also show some limitations. With the vast developments in battery electrolytes, there remains significant interest in tracking and understanding ion dynamics in such systems.^24,25^ Most SECM works evaluating ion transfer have focused on using Hg microelectrodes.^11,26−29^ Liquid Hg electrodes show several advantages such as high ion transfer rates and good ion selectivity, but they do not perform well in concentrated electrolytes.^30−32^ Only recent applications of pulsed-based methods have enabled measurements in electrolytes up to 100 mM.^29,32^ Herein, we explore an alternative SECM concept for tracking ionic flux by using a redox mediator. SECM already frequently applies redox mediators for acquiring various information at electrode surfaces,^33^ but mediators have yet to be explored for alkali ion sensing applications to the best of our knowledge.

In the past, several reports have demonstrated a strong relationship between the redox response of ferro/ferricyanide ([Fe(CN)_6_]^4–^/[Fe(CN)_6_]^3–^) and the concentration of the supporting electrolyte.^34−36^ An intriguing phenomenon is an observed redox potential shift of the [Fe(CN)_6_]^4–^/[Fe(CN)_6_]^3–^ couple as a function of the concentration of the supporting electrolyte, i.e., ionic strength of the electrolyte solution, showing a linear relationship with log concentration for several cations relevant to energy storage. This behavior does not follow the simple Debye–Hückel case, mostly likely due to incomplete dissociation or clustering between the ferri/ferrocyanide species and the electrolyte ions at high electrolyte concentrations.^34,37,38^ Similar shifts in the redox potential have also been demonstrated with microelectrodes in concentrations >1 M.^36^ Such concentrations are highly amenable for typical battery conditions and significantly closer to the highly concentrated electrolytes being explored for high-voltage aqueous batteries.^39,40^ Herein, we explore the usage of the *E*_1/2_ shift of the ferrocyanide species as a redox probe for in situ tracking of interfacial ion transfer. Through linear sweep voltammetry (LSV) measurements at a Pt microelectrode (PtUME), we found a reproducible and linear shift of the *E*_1/2_ between 0.5 and 3 M [K^+^]. We further combined the LSV measurements with SECM to track the in situ uptake and release of K^+^ at an operating composite electrode. Comparing the estimated concentration change based on the *E*_1/2_ shift with COMSOL modeling enabled quantification of the ion transfer kinetics.

## Experimental Section

### Electrolyte Preparation

Electrolyte solutions were prepared by mixing potassium chloride (KCl, Wako), potassium bis(fluorosulfonyl)amide (K[N(SO_2_F)_2_], KFSA, Solvionic), or lithium bis(fluorosulfonyl)amide (Li[N(SO_2_F)_2_], LiFSA, Kanto Chemical), with deionized water at room temperature. For all ion measurements, potassium hexacyanoferrate(II) trihydrate (K_4_[Fe(CN_6_)]·3H_2_O, Kanto Chemical) or potassium hexacyanoferrate(III) (K_3_[Fe(CN_6_)], Kanto Chemical) were further dissolved in the electrolytes.

### Electrode Preparation

Platinum ultramicroelectrodes (PtUMEs) were prepared as reported elsewhere^19^ using Pt microwires (20 μm diameter, Nilaco). Before each experiment, the PtUME was polished using 1.0 μm alumina (MicroPolish Alumina, Buehler) on a felt pad. Battery electrodes were prepared using 80 wt % 3,4,9,10-perylenetetracarboxylic diimide (PTCDI, Combi Blocks) as the active material, 10 wt % acetylene black (AB, Li250, Denka) as a conductive additive, and 10 wt % PVdF (#9100, Kureha) as the binder. First, the PTCDI and AB were manually mixed with a mortar and pestle then added to PVdF dissolved in NMP. The mixture was made into a uniform slurry using a planetary mixer (ARE-310, Thinky) for ∼20 min, spread onto Ti foil using a doctor blade, then dried at 80 °C under vacuum. Alternatively, thicker electrodes were prepared by replacing the PVdF with a 10 wt % polytetrafluoroethylene binder (F-104, Daikin). In this case, the electrodes were mixed to make a thick electrode mass. Thereafter, the mass was rolled flat and cut into 10 mm discs. The discs were pressed into a Ti mesh using a hydraulic press.

### Electrochemical Measurements

All electrochemical measurements were conducted using either a HZ-5000 potentiostat (Hokuto Denko Corp.) or a DY2323 bipotentiostat (ALS). Linear sweep voltammetry (LSV) measurements were conducted using a Ag/AgCl reference electrode (RE2B, EC FRONTIER) and a Pt wire as the counter electrode. For acquiring each calibration curve, concentrated electrolytes were prepared with an added 20 mM K_4_[Fe(CN_6_)] or K_3_[Fe(CN_6_)]. An LSV was collected with a PtUME at a scan rate of 50 mV/s between 0.1 and 0.6 V vs Ag/AgCl. After collecting the LSV, the K^+^ concentration was diluted using a mediator solution containing no added supporting electrolyte. After each addition, the solution was briefly stirred with a magnetic stir bar, and then another LSV was collected before further dilution. For reduction of [Fe(CN_6_)]^3–^, the electrochemical cell was bubbled with N_2_ prior to the measurement to avoid reduction of atmospheric oxygen at the PtUME.

For scanning electrochemical (SECM) measurements, we used an in-lab-constructed SECM setup and a modified three-electrode cell (SB1a; EC Frontier) as described in our previous report.^19^ The cell was covered with Parafilm© and bubbled with N_2_, then left above the solution during SECM measurements to minimize O_2_ entering the cell. We positioned the PtUME by applying a constant potential (0.6 V vs Ag/AgCl) to continuously oxidize [Fe(CN_6_)]^4–^ while moving it toward the PTCDI surface. After observing feedback, the electrode was manually stopped, and the response was fitted for determination of the approximate distance between the PtUME and PTCDI electrodes. While maintaining the same PtUME–PTCDI distance, LSVs were continually collected at the PtUME while applying potential steps at the PTCDI electrode.

Bulk voltammetry measurements of PTCDI were also conducted using a three-electrode cell (SB1a, EC Frontier). We used activated carbon as the counter electrode and Ag/AgCl as the reference electrode.

### Simulations

Simulations were conducted with COMSOL Multiphysics 6.1 using the Transport of Diluted Species module. Our simulations involved a 2D axisymmetric geometry containing a microelectrode surface, a battery electrode domain, and a solution domain which represent the electrode configuration during the SECM measurement.^11,30^ Fick’s laws were used to govern diffusion. The battery electrode domain was modeled as a porous medium, incorporating both porosity and tortuosity parameters. For physical parameters, we used both experimental and reported values, as noted in Tables S1 and S2. To evaluate the ion uptake process and extract interfacial ion transfer kinetics, we simulated the uptake of K^+^ using various forward rates, *k*_fK+_. Comparing the simulations with our sampling time (when the LSV occurred after the potential step) enabled an estimate of the local *k*_fK+_. To further extract a standard rate,  the simulations were repeated in succession with increasing overpotential. The increasing overpotential was related to *k*_fK+_ using the Butler–Volmer formulation of electrode kinetics.^41^ Using the results from each previous simulation as the starting conditions, we could predict the expected changes in K^+^ concentrations during each potential step and estimate a final . For the PtUME, we simulated a surface probe to evaluate changes in [K^+^] at a given PtUME–PTCDI distance during the K^+^ insertion process. The simulated changes in the concentration were then compared with the experimental data by using the time point of the measurement and the expected concentration change based on the calibration curve.

## Results and Discussion

**Figure 1:**
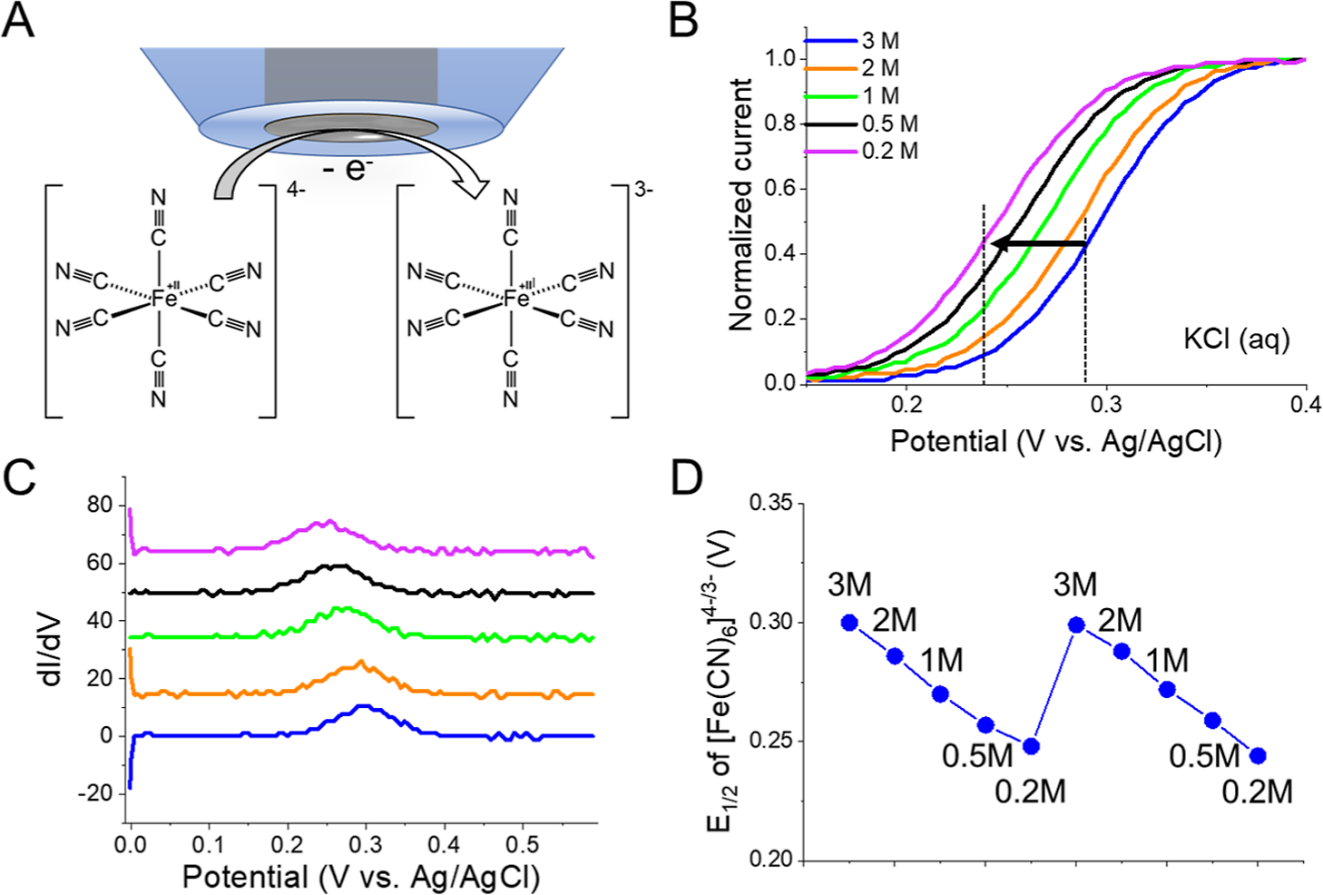
Utilizing ([Fe(CN)_6_]^4–^) oxidation at a PtUME to detect changing K^+^ concentrations. (A) Scheme of the electrochemical reaction of ([Fe(CN)_6_]^4–^) at a PtUME. (B) LSVs of ([Fe(CN)_6_]^4–^) oxidation at different K^+^ concentrations. (C) First derivative plots of the LSVs collected in (B). (D) Change in *E*_1/2_ during stepwise dilution of a KCl electrolyte containing 20 mM [Fe(CN)_6_]^4–^.

To explore the usage of ferro/ferricyanide for ion sensing measurements, we evaluated the oxidation of ferrocyanide at a Pt ultramicroelectrode (PtUME) (Figure 1A) in different concentrations of KCl. Starting with a 3 M solution of KCl containing 20 mM ferrocyanide ([Fe(CN)_6_]^4–^), we collected an LSV by sweeping the PtUME from the open circuit potential (OCP) toward oxidizing potentials and observed a standard sigmoidal response (Figure 1B). We diluted the KCl concentration sequentially and continued to collect LSVs at each concentration. As the K^+^ concentration was decreased, we observed a negative shift in the measured linear sweep voltammogram (LSV) and its associated half-wave potential (*E*_1/2_) (Figure 1B). Alternatively, this can be thought of as a positive shift with increasing [K^+^] or activity of K^+^. This shift could be clearly visualized with the first derivative plot of the LSV that provides a peak at the inflection point, which is close in value to the *E*_1/2_ (Figure 1C),^42^ and so, we will refer to this inflection potential as the *E*_1/2_. Moving the PtUME back to higher [K^+^] concentrations returned to the original measured *E*_1/2_ (within 3 mV) and the same shift was observed when diluting the electrolyte again (Figure 1D).

**Figure 2:**
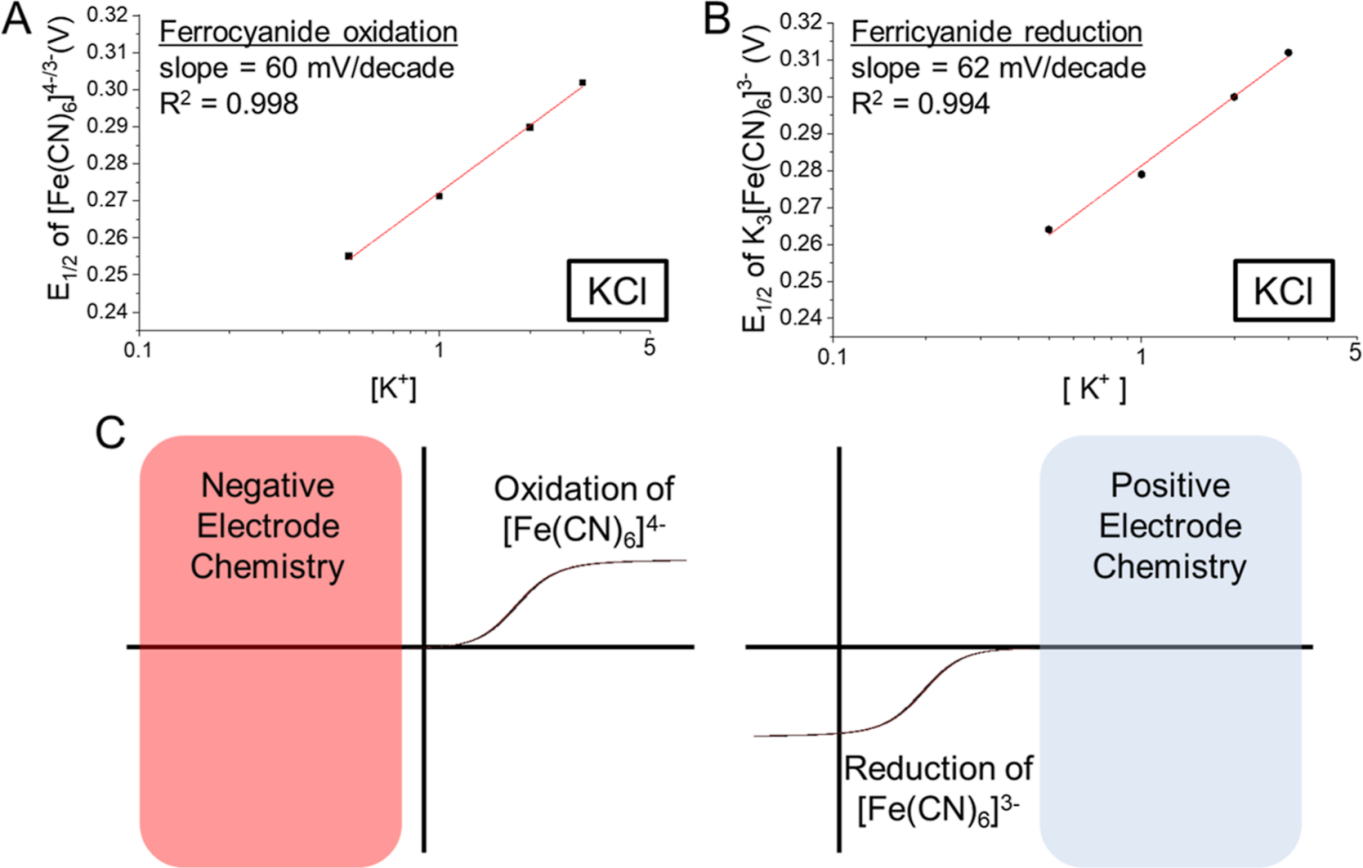
*E*_1/2_ shift of [Fe(CN)_6_]^4–/3–^ for battery analysis. *E*_1/2_ changes extracted from (A) K_4_[Fe(CN)_6_] and (B) K_3_[Fe(CN)_6_] in KCl. (C) Use of [Fe(CN)_6_]^4–^ oxidation and [Fe(CN)_6_]^3–^ reduction for evaluating negative and positive electrode chemistries in aqueous batteries.

Comparing the *E*_1/2_ shift to the log [K^+^] showed a good linear relationship (*R*^2^ = 0.998) between 0.5 and 3 M with a slope of 60 mV per decade (Figure 2A). When collecting consecutive measurements, we found the error in the LSV to be negligible (Figure S1) except for at low concentrations (e.g., 0.2 M). On the other hand, some changes in the slope were observed on different days (Figure S2). Like a pH meter, the slope should change with differences in the temperature as well as our microelectrode condition. Ideally, calibration should be done soon before the analysis or under controlled environmental conditions with the same microelectrode. For the highest accuracy, many measurements should be conducted, but only three measurements are typically employed for pH meters which span several orders of magnitude.

Considering the origin of the shift, [Fe(CN)_6_]^3–^ and [Fe(CN)_6_]^4–^ are thought to behave as strong electrolytes where all the K^+^ ions become dissociated. This leads to electrochemical behavior and activities that are highly sensitive to the supporting electrolyte concentration.^34,35^ For the one electron ferro/ferricyanide redox reaction of eq 1 occurring at the microelectrode
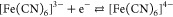
1we can expect an approximate relationship between the *E*_1/2_ and activities of the redox couple, similar to the Nernst form
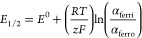
2where *E*^0^ is the standard reduction potential, *R*, *T*, *z*, and *F* are standard constants, and α_ferro_ and α_ferri_ are the activities of [Fe(CN)_6_]^4–^ and [Fe(CN)_6_]^3–^, respectively.

As [K^+^] increases, both α_ferro_ and α_ferri_ decrease due to their interaction with the higher K^+^ content in solution.^34,35^ However, the higher negative charge on [Fe(CN)_6_]^4–^ leads to a stronger interaction with K^+^ compared with [Fe(CN)_6_]^3–^, and thus we can expect α_ferro_ to decrease more rapidly than α_ferri_ with increasing [K^+^]. Thus, eq 2 predicts that higher ionic strengths can lead to an increase in α_ferri_/α_ferro_ and a positive shift in the *E*_1/2_ and vice versa. We note the response does not show good linearity with the square root of ionic strength, i.e., Debye–Hückel theory; so, the impact of K^+^ on the activity of the redox mediator is a more complex situation. A previous theoretical work on the ferrocene/ferrocenium redox couple likewise predicts a similar change in the redox potential, which is attributed to ion-pairing and activity.^38^ Some other evidence also suggests ion pairing with ferri/ferrocyanide.^43^ This seems reasonable considering the high negative charge states of the [Fe(CN)_6_]^4–^ and [Fe(CN)_6_]^3–^ complexes. Furthermore, when we tested the LSV behavior at low electrolyte concentrations, below 200 mM, we observed a shifting back toward the positive direction and more notable changes in kinetics (Figure S3). As with the *E*_1/2_, the kinetics also change with electrolyte concentrations,^35^ but this is likely already encompassed within the *E*_1/2_ shift while collecting the calibration curve. Microelectrodes provide the interesting advantage of stable operation with low or no added electrolyte, but this does not appear to enable ion sensing under such conditions.

The reverse process, or reduction of [Fe(CN)_6_]^3–^, also showed a linear shift (*R*^2^ = 0.99) with a similar slope of ∼62 mV across the same concentration range (Figures 2B and S4). As with the oxidation process, we could reproducibly observe shifting of the measured *E*_1/2_ with changes in concentrations, but we again emphasize that the slope will change with experimental or environmental conditions. Nevertheless, these results are quite promising for analytical purposes because oxidation of [Fe(CN)_6_]^4–^ cannot be used to evaluate cathode materials with a redox potential more positive than [Fe(CN)_6_]^4–^. So, reduction of [Fe(CN)_6_]^3–^ or oxidation of [Fe(CN)_6_]^4–^ can be used to evaluate ion transfer at positive and negative battery electrodes, respectively (Figure 1C).

Beyond KCl, we also evaluated the concentration dependence with the common energy storage counterion, bis(fluorosulfonyl)amide (FSA^–^) for both K^+^ and Li^+^ near the 1 M concentration range. *Caution* should be used when working with the FSA^–^ salts in aqueous electrolytes as they can decompose to form hydrofluoric acid, HF, especially when prepared at high concentrations.^44^ In the case of KFSA, we observed similar shifting in the *E*_1/2_ and good reproducibility, but the *R*^2^ (0.978) was a bit low (Figure S5). The impact of the anion on [Fe(CN)_6_]^4–/3–^ activity has been reported previously,^34,35^ and we suspect the anion should play a role in the behavior of [Fe(CN)_6_]^4–/3–^ as it should impact solubility, solution structure, etc. It is difficult to differentiate the role of these individual species using our methods. Furthermore, there may be an unknown impact of the junction potential when in contact with the Ag/AgCl reference electrode. These are interesting points to consider for future studies. In contrast to KFSA, LiFSA showed a good linear dependence (*R*^2^ = 0.995) though the slope change was notably larger (Figure S6). The solutions were prepared fresh, but some decomposition may contribute to this difference.^44^ Still, a stronger *E*_1/2_ shift in other aqueous Li^+^ electrolytes has been reported, and linked to differences in solubility for Li^+^, K^+^, etc.^45^ The use of a [Fe(CN)]_6_]^4–/3–^ redox mediator with a microelectrode seems to be as versatile as in previous reports and a plausible concept for estimating local ion concentration changes at operating battery electrodes.

**Figure 3:**
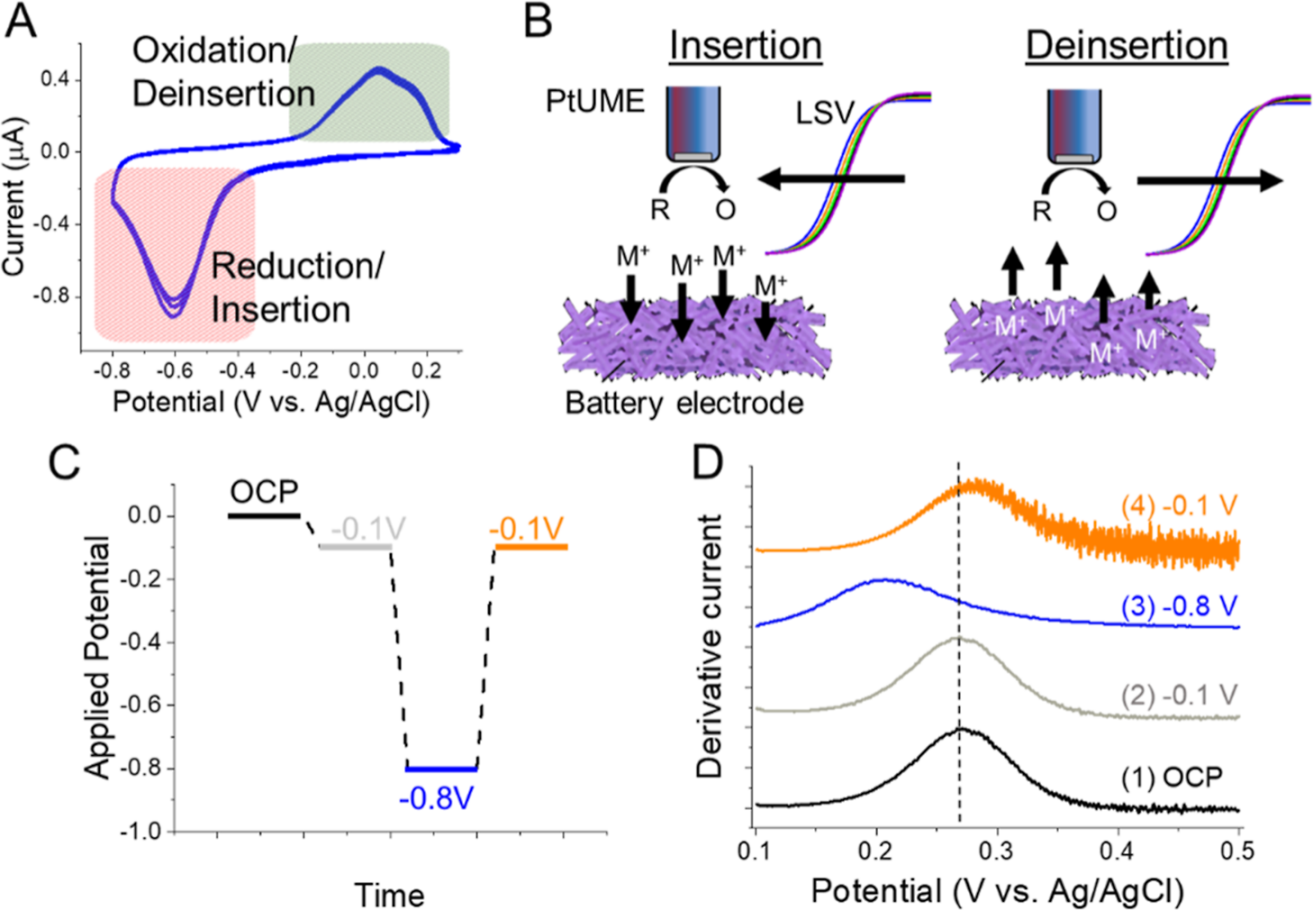
Detecting changing K^+^ concentrations at a battery electrode. (A) CV response of PTCDI electrode in 1 M KCl. (B) Concept of using redox mediator for ion sensing. (C) Potential step pattern used to evaluate ion uptake at PTCDI. (D) First derivative response of the PtUME during potential steps at PTCDI.

To evaluate changes in the ion concentration for battery applications, we focused on an operating composite PTCDI electrode. PTCDI is an organic material recently explored as a high-rate negative electrode for various aqueous batteries.^39,46−48^ When using a 1 M K^+^ aqueous electrolyte solution, similar to the electrolyte concentrations found in commercial LIBs (1 M) and emerging K-ion and Na-ion batteries (0.75–1 M),^49,50^ we observed reversible oxidation/reduction peaks for PTCDI (Figure 3A). The large cathodic peak indicates reduction of the carbonyl groups and simultaneous insertion of K^+^ into the PTCDI structure. As this process occurs, the concentration of K^+^ is expected to decrease in the vicinity of the electrode surface which would lead to a negative shift in the [Fe(CN)_6_]^4–^ voltammogram (Figure 3B). Likewise, the anodic peak involves deinsertion of K^+^ ions from the PTCDI host, which would lead to an increase in [K^+^] near the electrode surface.^47,51^ We further note the large peak separation in the CV of PTCDI, which has also been observed in other aqueous and organic electrolytes.^47,48^ The peak splitting tended to increase with the scan rate (Figure S7), which can be an indication of kinetic limitations.^52,53^ However, as with polarization in batteries, the peak splitting can also reflect characteristics of the composite electrode (e.g., particle size, thickness, etc.).^54−56^

For investigating local changes in the K^+^ concentration at PTCDI, we started by examining the impact of a single potential step on the uptake of K^+^ and response at the PtUME. We approached and positioned the PtUME ∼ 11 μm above the surface of a PTCDI electrode using approach curve methods (Figure S8).^33,57,58^ Maintaining a constant PtUME-to-PTCDI distance, we collected continuous voltammetry measurements at the PtUME while applying a potentiostatic pulse (−0.8 V vs Ag/AgCl) at the PTCDI electrode (Figure 3C). Due to the thickness and resistance of the electrode (∼1 mm including the mesh current collector) as well as the large potential step, we observed apparent cross-talk issues during initial CV cycles (Figure S9). Cross-talk is an issue related to the bipotentiostat design and happens when there are large faradaic impedances or currents, or when there is high solution resistance.^59,60^ Under these conditions, the applied potential at the substrate electrode (PTCDI) impacts the potential and measured currents of the second electrode, the microelectrode. Because this predominantly occurs at short times, we focused only on the last CV cycle when the PTCDI current had substantially decreased and cross-talk should be minimized (i.e., the forward LSV collected ∼65 s after the potential step). Starting from OCP (Figure 3C,D), we stepped the PTCDI electrode potential to −0.1 V where no insertion occurs. In this case, the *E*_1/2_ shift in the measured voltammetry was negligible. When the PTCDI potential was stepped more negative to −0.8 V to initiate ion uptake, we could observe a large negative shift of ∼60 mV, blue curve in Figure 3D. Based on our calibration plot, these results suggest a large decrease in [K^+^] to <100 mM beneath the PtUME. As discussed earlier, there are some issues with evaluating the ion concentration at such low concentrations. The estimate can contain some error in this case, but adjusting the PtUME-to-PTCDI distance, potential step size, or step time can help alleviate this limitation.

**Figure 4:**
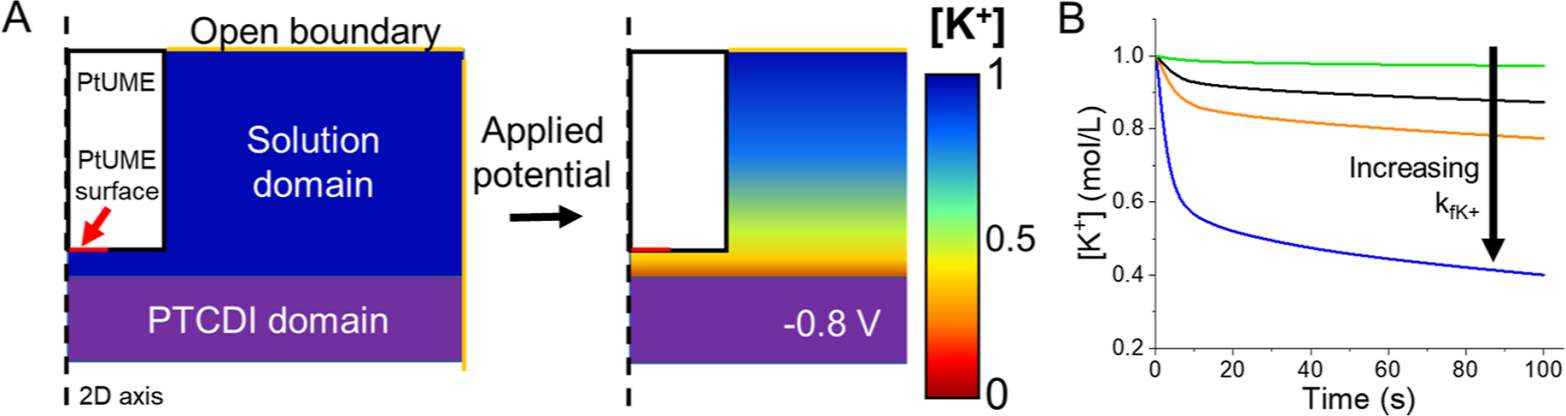
COMSOL simulation of K^+^ insertion. (A) 2D axisymmetric model and illustration of the concentration gradient during polarization. (B) Impact of *k*_fK+_ on measured [K^+^] at the PtUME surface.

**Figure 5:**
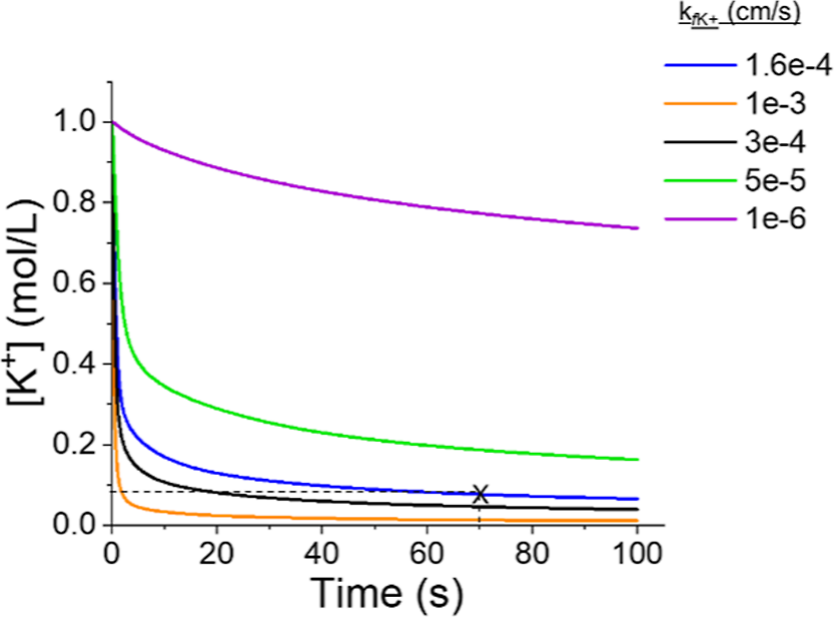
Fitting of experimental result to COMSOL simulations for K^+^ insertion. The x symbol marks the time and measured [K^+^] during a −800 mV potential step.

To further understand the large change in [K^+^] at the electrode surface, we built a 2D axisymmetric model in COMSOL Multiphysics (Figure 4A) alike to previous reports.^11,16^ Further details are provided in the Supporting Information, as listed in Tables S1 and S2. Through our model, we simulated the PTCDI composite electrode behavior and the impact of a surface insertion rate, *k*_fK+_ (cm/s), on the changes in the local ion concentration near the PtUME. The PTCDI electrode was modeled as a porous domain that consumes K^+^ ions at a given k_*f*K+_. For the PtUME, we used a simple concentration probe to evaluate the expected changes in [K^+^] at a given PtUME–PTCDI distance. As the simulation progresses, a diffusion gradient will develop at the PTCDI surface (illustrated in Figure 4A), and this is detected by the PtUME (Figure 4B). In reality, there can also be the development of a concentration gradient within the composite electrode, which is included in our simulations (Figure S10). On the other hand, the anions should migrate away from the electrode surface during polarization and K^+^ insertion, but this is not accounted for in our current simulations. As *k*_fK+_ is increased, more pronounced K^+^ depletion occurs at the surface. At slow rates, the PtUME needs to be positioned closer to the PTCDI or no change in the concentration can be detected. In the case of Figure 3D above, our simulations predict *k*_fK+_ of ∼1.6 × 10^–4^ cm/s for K^+^ insertion at the PTCDI surface (Figure 5 and Table S1). Such rates have been observed at electrode materials using other ion-sensitive SECM measurements.^11^ However, the usage of a large potential step, thickness of the electrode, as well as error in the PtUME-to-PTCDI distance could lead to some inaccuracy. In other words, suitable conditions of the potential step, electrode thickness, and distance are important for accurate tracking of [K^+^].

**Figure 6:**
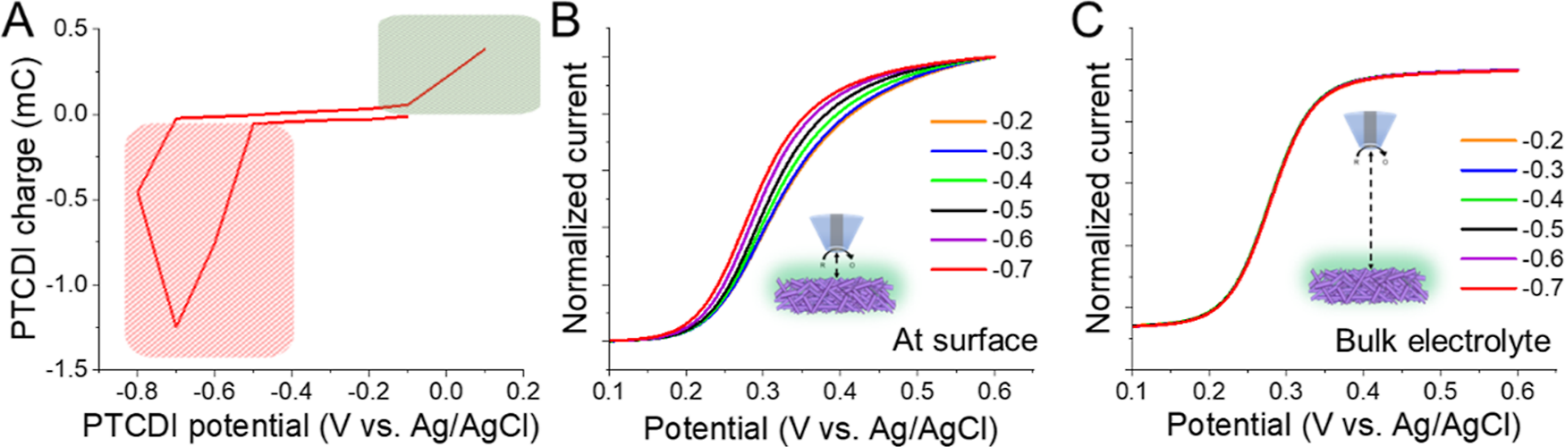
Detecting K^+^ de/insertion in 1 M [K^+^] solution. (A) Integrated response of PTCDI electrode during potential-step cycling. (B) Normalized LSVs of ([Fe(CN)_6_]^4–^) oxidation during reduction of PTCDI. (C) LSVs collected during reduction at a PtUME-to-PTCDI distance of 1000 μm.

Beyond determining the rate at a particular overpotential, our methods also provide means to attain standard rate constants, , by employing a simple kinetic model such as Butler–Volmer. In this way, the overpotential can be related to *k*_fK+_ based on the *E*^0^ and applied potential at PTCDI. For this, we used a thinner PTCDI electrode (∼10 μm thickness) to improve conductivity and minimize cross-talk during our measurements. We positioned the PtUME 7.6 μm from the PTCDI surface (Figure S11) and applied smaller potential steps of 100 mV between 0.1 and −0.8 V vs Ag/AgCl (Figure S12). The measurement time of each LSV was ∼35 s (equivalent scan rate for PTCDI: ∼3 mV/s). The integrated response of the PTCDI electrode shown in Figure 6A resembles the voltammetric response, though we could not access the full oxidation peak due to its overlap with [Fe(CN)_6_]^4–^ oxidation. As the potential of the PTCDI became more negative initiating ion insertion (red region of Figure 6A), we observed a notable *E*_1/2_ shift in the LSV measurements when the PtUME is positioned near the surface (Figure 6B). On the other hand, no notable shifts were observed when the microelectrode was left at distances far (1000 μm) from the PTCDI surface (Figure 6C), indicating the process was localized to the surface of the electrode and small perturbations did not induce any cross-talk.

**Figure 7:**
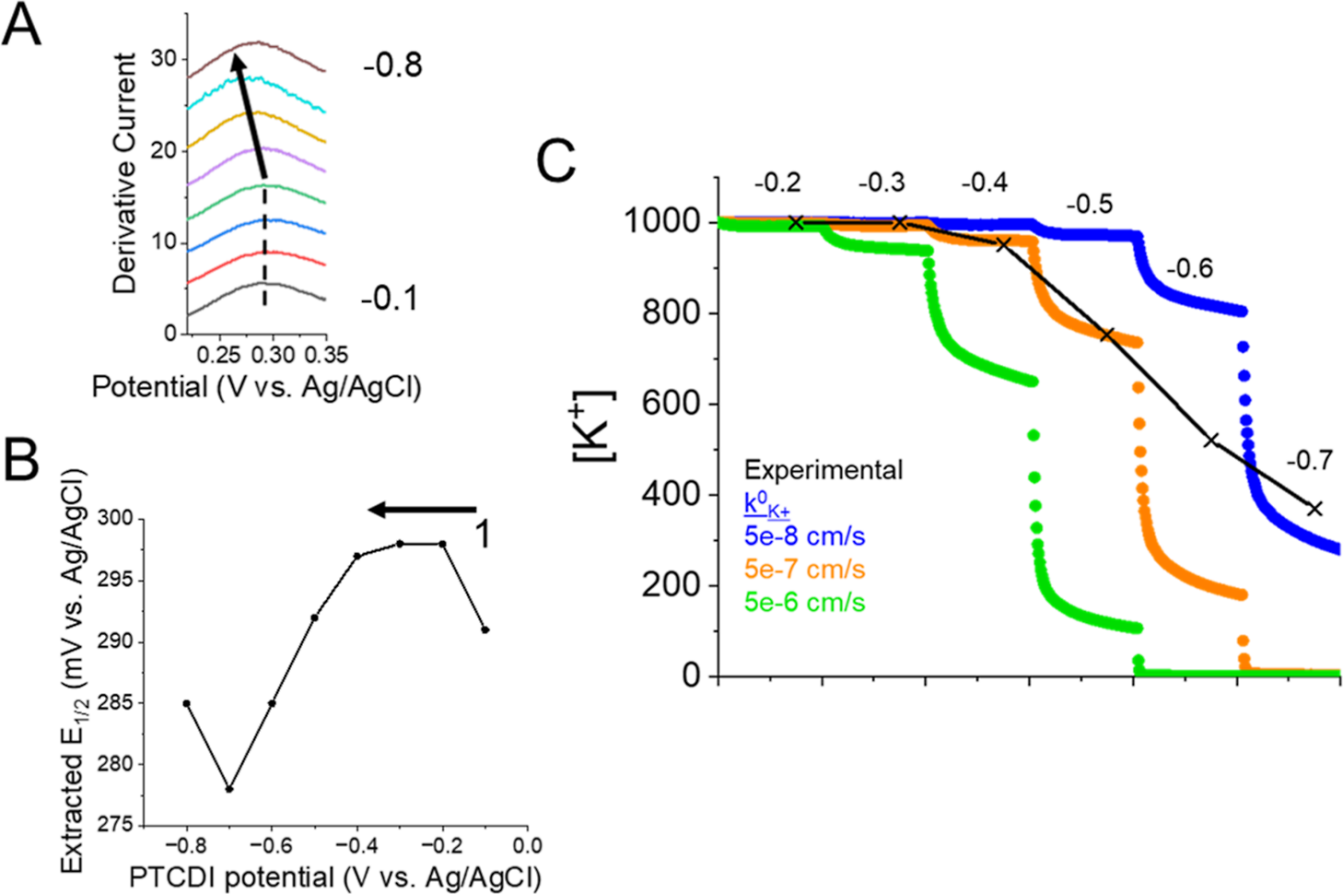
K^+^ insertion in 1 M [K^+^] electrolyte solution. (A) Plot of 1st derivative of LSVs and (B) extracted *E*_1/2_ during reduction of PTCDI. (C) Fitting of PtUME response to COMSOL simulations. The x symbols mark the estimated [K^+^] from the experimental data during each potential step.

Looking at the *E*_1/2_ shift during reduction (Figure 7A, 7B), we see a relatively stable *E*_1/2_ from −0.2 to −0.4 V, prior to K^+^ insertion. Thereafter, the *E*_1/2_ gradually shifted in the negative direction from −0.4 to −0.7 V. The shift suggests a growing K^+^ concentration gradient at the PTCDI surface during each potential step. The total shift was ∼21 mV or a decrease to 0.55 M under the experimental conditions (Table S2). We turned to our COMSOL model to further interpret the changes in ion concentration near the PtUME. In this case, we simulated the cumulative decrease in ion concentration for various  (Figure 7C). The simulations indicated a  on the order 10^–7^ cm/s. This is actually quite slow compared with electron transfer rates to organic redox species, which tend to be on the order of 10^–3^ cm/s or faster.^61^ Therefore, we believe the measured  in this case, is more appropriately an effective or apparent *k*^0^ for ion insertion at the composite electrode. The composite electrode structure will always impact the performance and rate capabilities. Our method provides means for understanding and quantifying such kinetic limits at composite electrodes.

**Figure 8:**
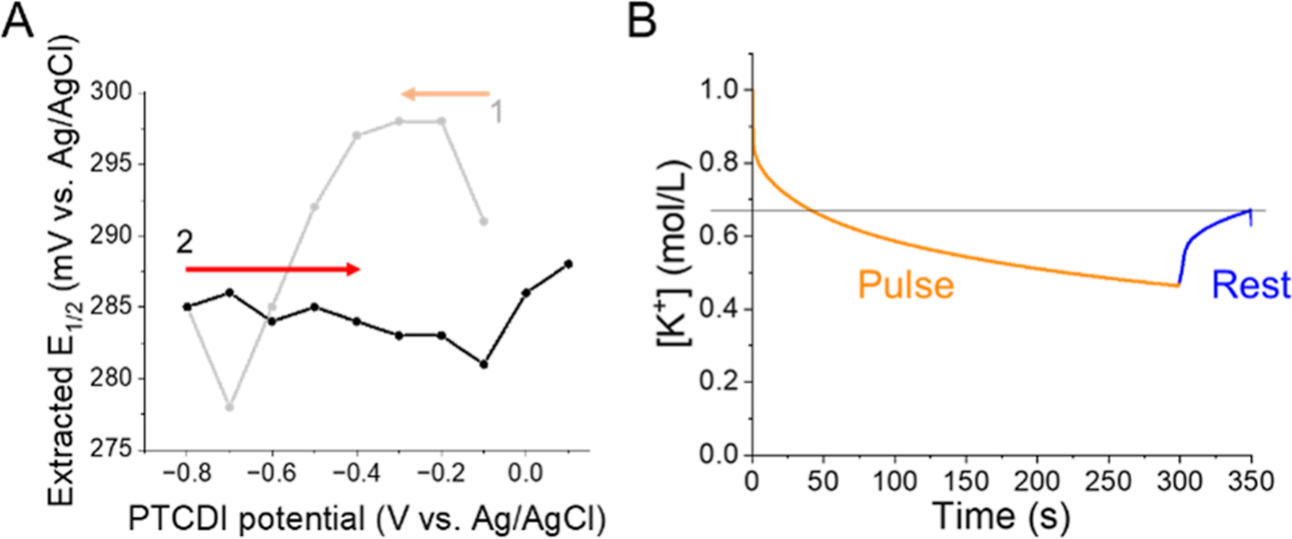
K^+^ extraction in 1 M [K^+^]. (A) Measured *E*_1/2_ during oxidation of PTCDI. (B) Modeling of K^+^ recovery after a potential step followed by a rest period with the electrode at open circuit.

When the PTCDI electrode potential was stepped back in the positive direction, we observed that the apparent ion concentration remained low between the PtUME and PTCDI surface (Figure 8A). From −0.7 to −0.1 V vs Ag/AgCl, we did not observe any release of the K^+^, and so the *E*_1/2_ remained shifted to potentials ∼285 mV with some associated drift. When the PTCDI electrode is at rest or there is no de/insertion chemistry occurring, changes in [K^+^] at the electrode surface are slow and limited to diffusion from the bulk electrolyte. Reaching equilibrium with the bulk electrolyte requires long times based on our simulations (Figure 8B). For example, [K^+^] would be expected to increase from 0.4 to 0.7 M after 50 s of rest. As the PTCDI electrode reached potentials positive to −0.1 V, the *E*_1/2_ started shifting in the positive direction indicating K^+^ extraction. Our methods seem to be effective for tracking such ion dynamics and could be combined with pulsing methods for ion insertion mapping.^16^

## Conclusions

In conclusion, we have demonstrated an in situ technique for evaluating ion transfer kinetics at operating battery electrodes, approaching high concentrations as found in common battery electrolytes. Our ion-sensitive methods build on SECM methodologies to utilize a redox mediator, ferro/ferricyanide, for evaluating changes in K^+^ concentrations. By tracking the linear shift in the measured *E*_1/2_ for both oxidation and reduction of ferro/ferricyanide, we could estimate changes in K^+^ in bulk electrolytes and at the surface of operating battery electrodes. Our methods seem to be broadly applicable for various counterions or when replacing K^+^ with other alkali and alkali earth cations such as Li^+^, Na^+^, Mg^2+^, and Ca^2+^. After positioning a PtUME near an operating PTCDI surface, we could detect gradual changes in the local [K^+^] during insertion and deinsertion processes. The rates of de/insertion were further quantified using COMSOL simulations. Based on our simulations, we observed an apparent insertion rate of ∼10^–7^ cm/s, which reflects the behavior of the composite electrode. While our methods are currently limited to aqueous conditions, other redox mediators also show shifting in their *E*_1/2_.^62,63^ As such, this phenomenon seems to have broad usage for various energy storage systems in future work.

## Supporting Information Available

Additional voltammetry, CV scan rate dependence of PTCDI, approach curve fittings, evidence of cross-talk issue, calibration curves, and simulation details (PDF)
