# Supplementary material for: Quantifying Interfacial Ion Transfer at Operating Potassium-Insertion Battery Electrodes within Highly Concentrated Aqueous Solutions
Source: ACS Appl Mater Interfaces. 2024 Jun 17;16(26):33379–87. doi: 10.1021/acsami.4c03645 (PMC11231980; doi:10.1021/acsami.4c03645)
Supplement: Supplementary file 1 — am4c03645_si_002.pdf [file am4c03645_si_002.pdf]

Supporting Information

Quantifying Interfacial Ion Transfer at Operating Potassium-Insertion Battery Electrodes within Highly Concentrated Aqueous Solutions

Zachary T. Gossage, Ryoichi Tatara, Tomooki Hosaka, Shinichi Komaba*

Department of Applied Chemistry, Tokyo University of Science, Tokyo 162-8601

Corresponding Author

* Email: komaba@rs.tus.ac.jp

Table of Contents page

Supplemental Figures and Tables

S1. Further LSVs of $[Fe(CN)_6]^{4-}$ oxidation in different KCl concentrations............... 3

S2. Calibration curve for $[Fe(CN)_6]^{4-}$ oxidation collected on a different day............. 4

S3. LSVs of $[Fe(CN)_6]^{4-}$ oxidation at low KCl concentrations.................................. 4

S4. Voltammetry of $[Fe(CN)_6]^{3-}$ reduction in different KCl concentrations 5

S5. Voltammetry of $[Fe(CN)_6]^{4-}$ oxidation in different KFSA concentrations 5

S6. Voltammetry of $[Fe(CN)_6]^{4-}$ oxidation in different LiFSA concentrations............ 6

S7. CV scan rate dependence of PTCDI composite electrode 6

S8. Approach curve and fitting to thick PTCDI composite electrode 7

S9. Cross-talk issue at thick PTCDI electrode.. 7

S10. Simulation of K^+ distribution during a potential step at PTCDI 8

S11. Approach curve and fitting to 10 µm PTCDI composite electrode................... 8

S12. Potential step used for in situ measurement at thin PTCDI electrode 9

Table S1. Simulation details for Experiment 1 (700 mV step) 9

Table S2. Simulation details for Experiment 2 (100 mV steps).............................. 10

References 10

Supplemental Figures and Tables

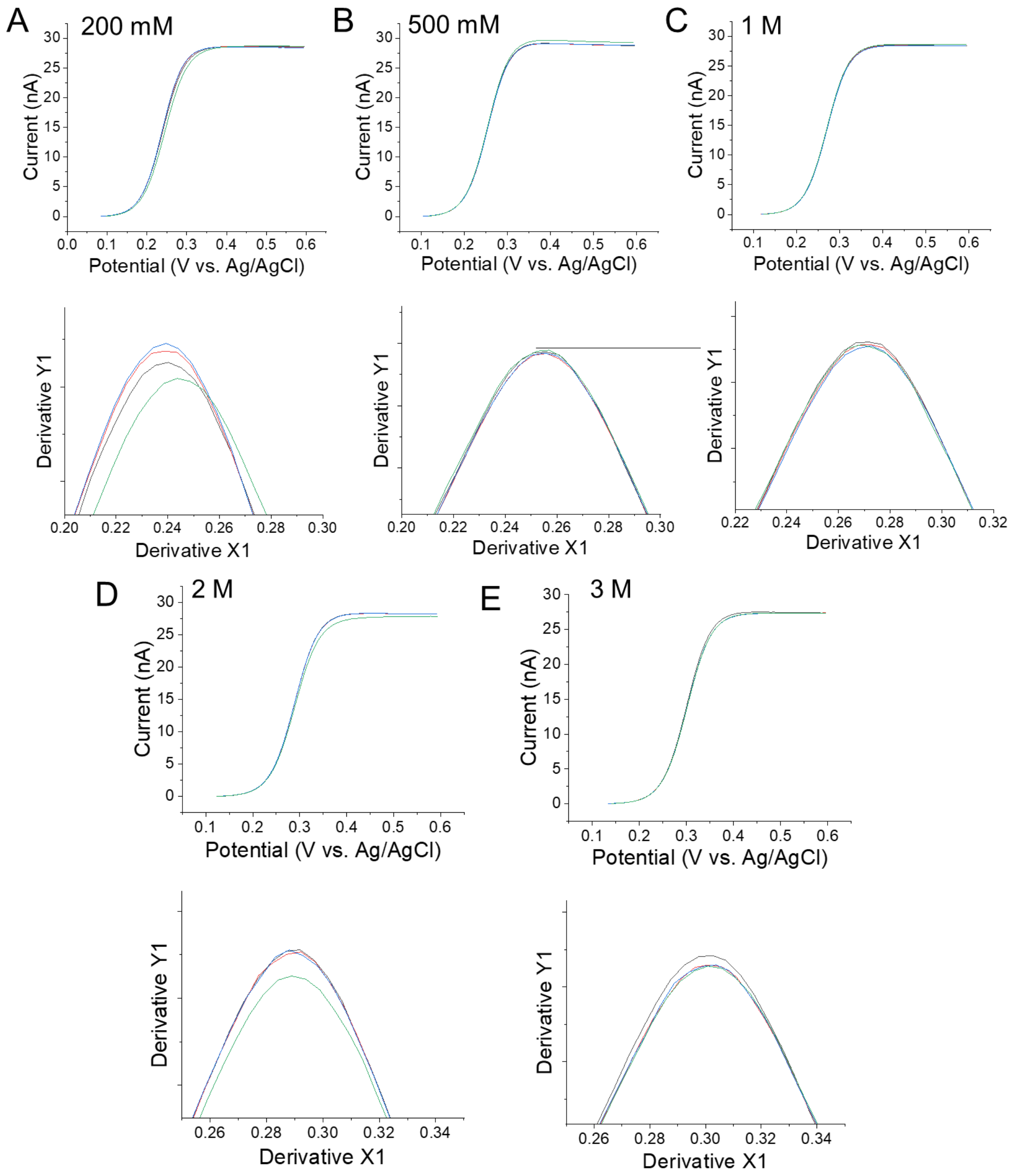

Figure S1. Sequential linear sweep voltammetry of $[Fe(CN)_6]^{3-}$ reduction in different K^+ concentrations. Each concentration shows 4 measurements. The K^+ salt used was KCl.

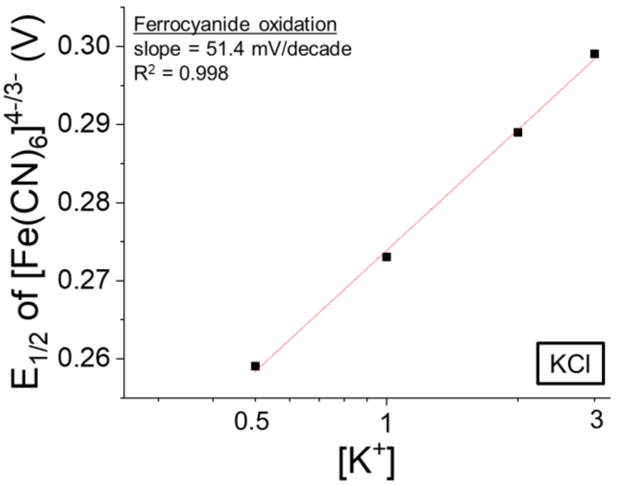

Figure S2. Calibration curve collected on a different day using $[Fe(CN)_6]^{4-}$ oxidation. The K^+ salt used was KCl.

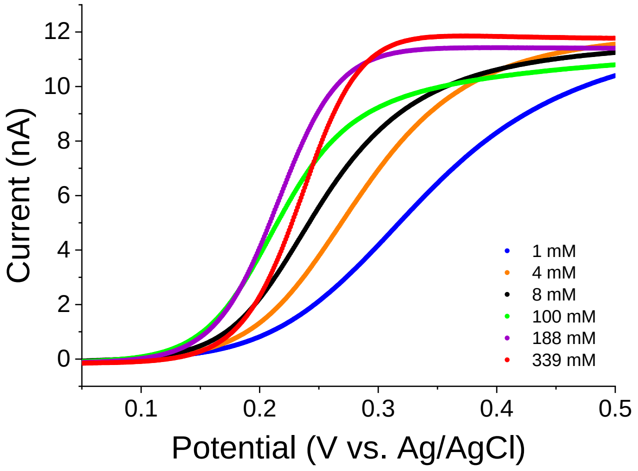

Figure S3. Linear sweep voltammetry of $[Fe(CN)_6]^{4-}$ oxidation at low K^+ concentrations. The K^+ salt used was KCl.

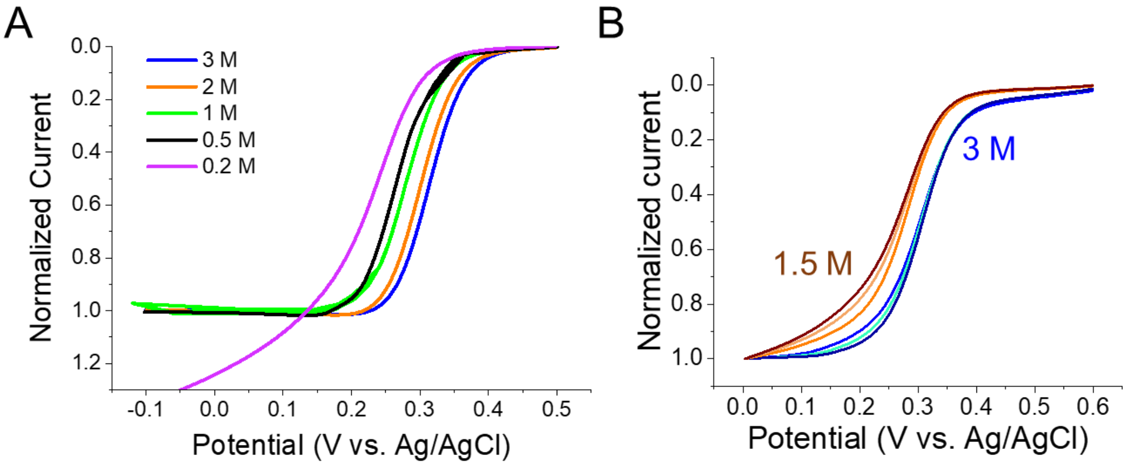

Figure S4. (A) Linear sweep voltammetry of $[Fe(CN)_6]^{3-}$ reduction in different K^+ concentrations. (B) Consistent $E_{1/2}$ shift for consecutive measurements at different concentrations. The K^+ salt used was KCl.

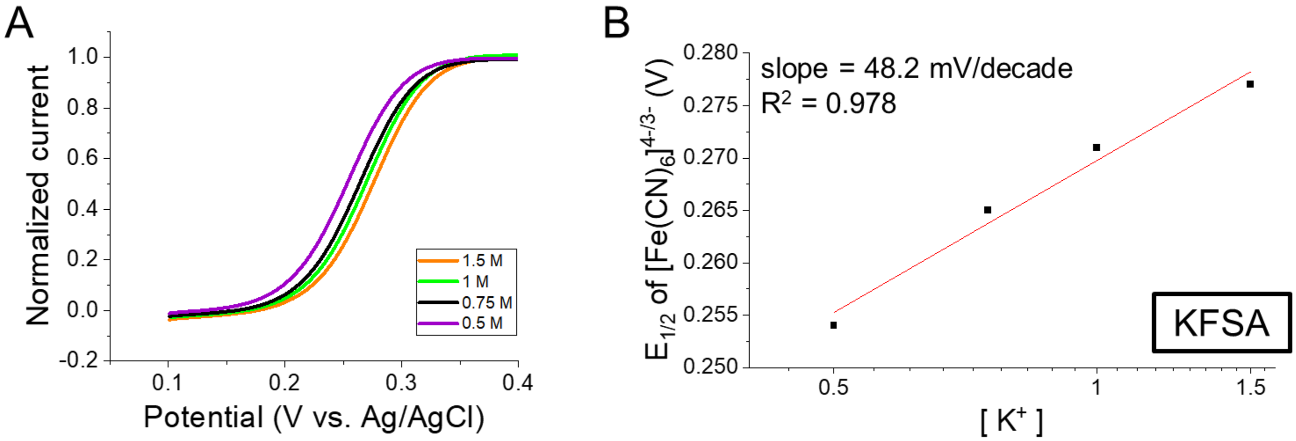

Figure S5. Linear sweep voltammetry and extracted $E_{1/2}$ for $[Fe(CN)_6]^{4-}$ oxidation in different K^+ concentrations. The K^+ salt used was KFSA.

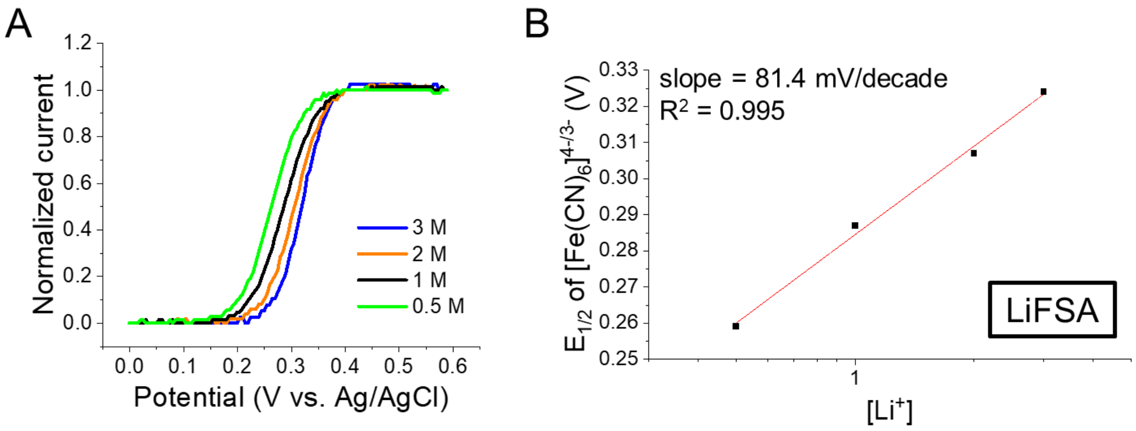

Figure S6. Linear sweep voltammetry and extracted $E_{1/2}$ for $[Fe(CN)_6]^{4-}$ oxidation in different Li^+ concentrations. The Li^+ salt used was LiFSA.

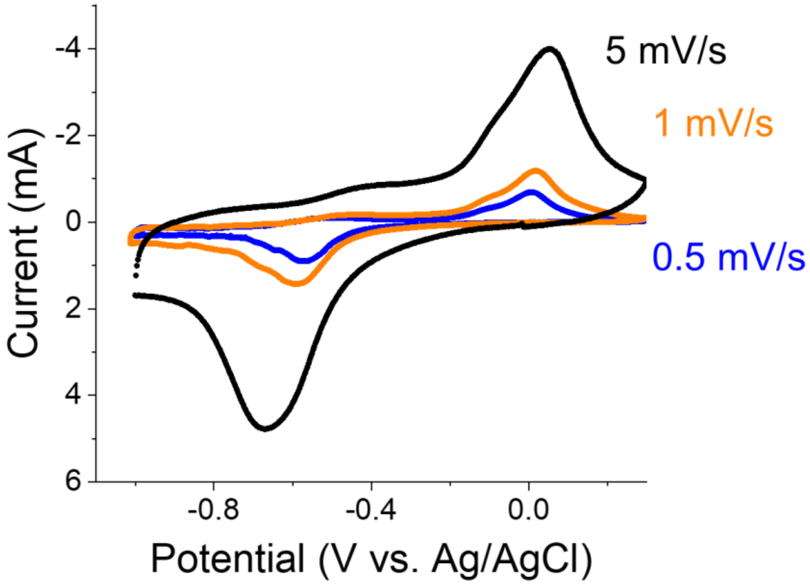

Figure S7. Scan rate dependence of PTCDI composite electrode. The electrode was cycled in 1 M KFSA in water.

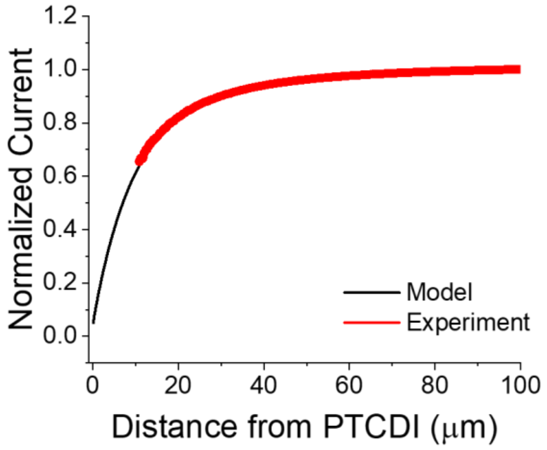

Figure S8. Probe approach curve and fitting to thick PTCDI/KB/PTFE composite electrode.

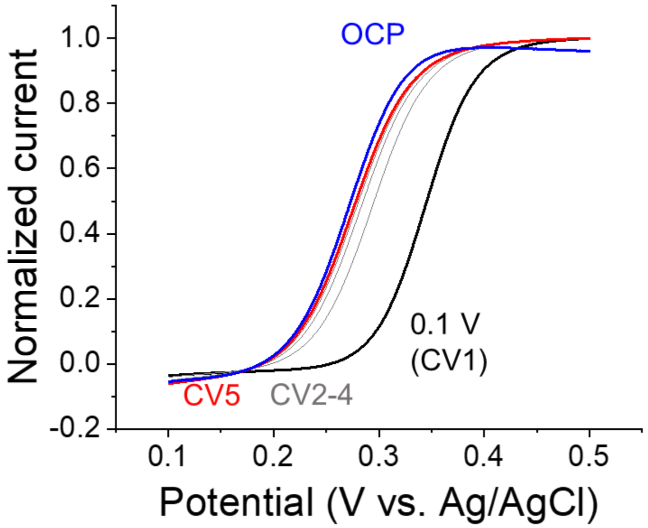

Figure S9. Cross-talk issue observed with probe retracted (1000 µm from thick PTCDI electrode). The CV cycles at the microelectrode, labeled CV1-5, were collected while applying a potential step to 0.1 V vs. Ag/AgCl (from OCP). The LSV collected at OCP is shown for reference.

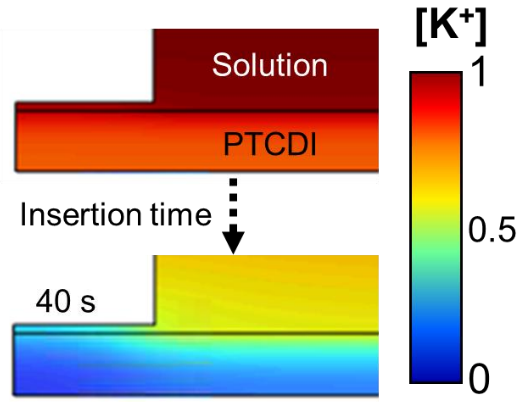

Figure S10. Simulation of K^+ distribution during a potential step at the PTCDI electrode.

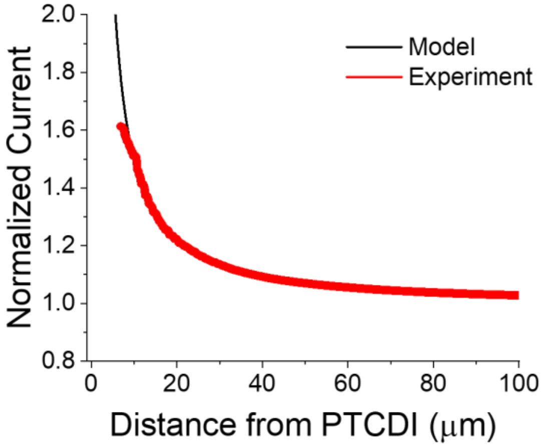

Figure S11. Probe approach curve and fitting to a 10 μm PTCDI/KB/PVDf composite electrode.

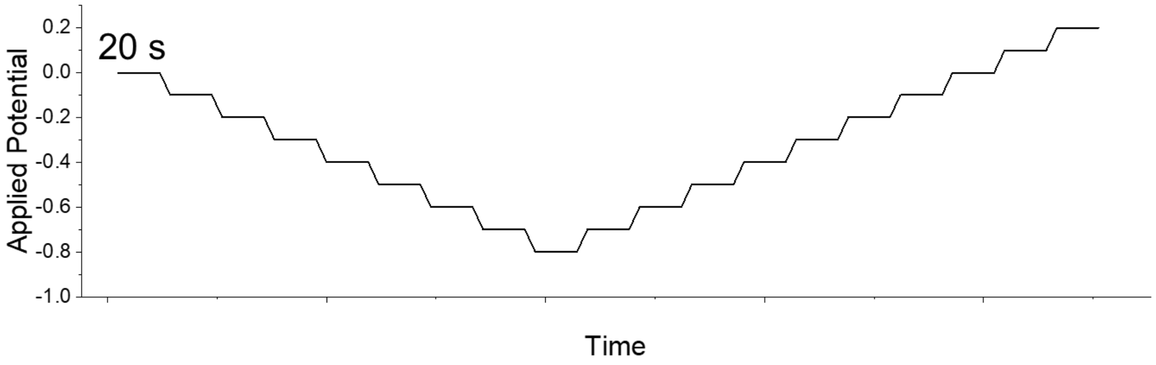

Figure S12. Potential steps used in controlling the PTCDI during the SECM measurement.

Table S1. COMSOL simulation parameters for thick PTCDI electrode

Parameter	Value	Unit	Reference
PtUME radius (a)	10	µm	
PtUME RG	8	(glass:electrode)	
PtUME-PTCDI distance	11	µm	
PTCDI domain thickness	1000	µm	
PTCDI porosity	27	%	
PTCDI tortuosity	4		1
Diffusion coefficient of K^+ (for bulk and in PTCDI domains)	1.89e-9	m^2/s	2
Initial K^+ concentration	1000	mol/m^3	
α (transfer coefficient)	0.5		
E^0 for PTCDI	-0.3	V vs. Ag/AgCl	
Time of measurement	70	s	

Table S2. COMSOL simulation parameters for thin PTCDI electrode

Parameter	Value	Unit	Reference
PtUME radius (a)	10	µm	
PtUME RG	8	(glass:electrode)	
PtUME-PTCDI distance	7.6	µm	
PTCDI domain thickness	10	µm	
PTCDI porosity	70	%	
PTCDI tortuosity	4		
Diffusion coefficient of K^+ (for bulk and in PTCDI domains)	1.89e-9	m^2/s	2
Initial K^+ concentration	1000	mol/m^3	
α (transfer coefficient)	0.5		
E^0 for PTCDI	-0.3	V vs. Ag/AgCl	
Time of measurement	15	s	

References

1 Kehrwald, Dirk, et al. "Local tortuosity inhomogeneities in a lithium battery composite electrode." Journal of The Electrochemical Society 158.12 (2011): A1393.

2 Secuianu, Catinca, et al. "Mutual diffusion coefficients of aqueous KCl at high pressures measured by the Taylor dispersion method." Journal of Chemical & Engineering Data 56.12 (2011): 4840-4848.
